# In this issue

**Published:** 2023-05

**Corticosteroids treatment for pediatric acute respiratory syndrome. *A critical review***

Al-Sofyani examines steroid use in treating pediatric acute respiratory distress (PARDS) syndrome, emphasizes current developments in the field, and gives a broad overview of PARDS management. Approximately 25% of all pediatric consultations are due to respiratory conditions, 10% of which are for asthma. Regarding prevalence, bronchiolitis, acute bronchitis, and respiratory infections are other leading pediatric respiratory illnesses. Compared to the aforementioned diseases, PARDS is rare but lethal in the intensive care unit patients.

***see page 440***

**Figure:**
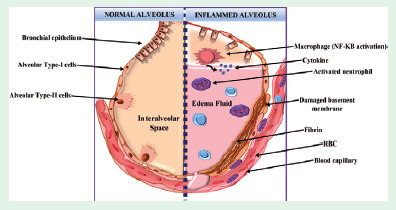
A healthy alveolus with intact alveolar cell components and the vascular epithelial membrane is shown in the left panel. Following an acute inflammatory insult, alveolar alterations are seen in the right panel.

