# Supplementary material for: In this issue
Source: Saudi Med J. 2023 May;44(5):429. (PMC10187738)
Supplement: Related texts

# Abstract: Original Articles

**Levels and profiles of pentabromodiphenyl ether contaminants in human breast milk from Riyadh, Saudi Arabia**

Yakout et al investigate levels of pentabromodiphenyl ether (PBDEs) ether in breast milk samples from healthy mothers (n=75) who had lived in Riyadh for the last 5 years. The significant levels of PBDEs that occur in the meat and poultry reared in Saudi Arabia need further investigation especially as Saudis among largest consumers of poultry meat. The mothers in this study consumed more meat, followed by the egg, and milk 36%. The majority of donors consumed fish and egg 2 times per week. The samples were collected and were extracted, cleaned by solid-phase extraction and PBDEs analysis was done using gas chromatography–mass spectrometry. All PBDE congeners were detected in the human breast milk samples with high detection frequency (98%). The dominant congener was BDE 47, accounting more than 39% of all BDE congeners, followed by BDE-99 and BDEs 153 which accounted for18% and 12% of the total BDE congeners respectively.

***see page 450***

**The prevalence and risk factors for hiatal hernia among patients undergoing endoscopy. *A retrospective analysis***

Alsahafi et al determine the prevalence of hiatal hernia (HH) and assess its association with gender, age, body mass index (BMI) among 2805 patients. The prevalence of HH was 28.9% based on this large endoscopy-based population. We found no association between HH and gender, age, or BMI. The mean age was 48.6 (±18.6) years and males constituted 28.8% of the study population. The mean BMI was 29.7 (±8.6) kg/m^2^. The prevalence of HH was 29.8% among all patients and 48.6% among those who underwent esophagogastroduodenoscopy for gastroesophageal reflux disease–related indications.

***see page 509***
